# Controlled Synthesis and Microstructure of Metastable Flower-Like Vaterite

**DOI:** 10.3390/ma11112300

**Published:** 2018-11-16

**Authors:** Yebin Guan, Xiaohong Wang, Weicheng Cao, Gentao Zhou

**Affiliations:** 1 Anhui Key Laboratory of Functional Coordination Compounds, School of Chemistry and Chemical Engineering, Anqing Normal University, Anqing 246011, China; guanyb@aqnu.edu.cn; 2 CAS Key Laboratory of Crust-Mantle Materials and Environments, School of Earth and Space Sciences, University of Science and Technology of China, Hefei 230026, China; 3 Center for Chemistry of Novel & High-Performance Materials, and Department of Chemistry, Zhejiang University, Hangzhou 310027, China; caowc@zju.edu.cn

**Keywords:** calcium carbonate, vaterite, microwave, 2-naphthaleneacetic acid (2-NAA)

## Abstract

Developing a simple morphology-controlled synthesis of metastable vaterite is a goal in the field of materials research. In this paper, we successfully synthesized flower-like dendritic vaterite crystals using a microwave method with 2-naphthaleneacetic acid (2-NAA) and ethylene glycol (EG) as the regulating additives. The results show that the morphology of vaterite could be regulated by inducing a monolayer or multilayer flower-like structure with the appropriate choice of regulators. Interestingly, the microstructure analysis showed that such flower-like vaterite dendrites host two different kinds of crystal cells. The negative carbonate 2-NAA effectively neutralized the charge of the vaterite (001) plane, resulting in the crystalline growth along the direction parallel to it and inducing a flower-like morphology. This experiment reveals an alternative approach to controlling hierarchical structures during the synthesis of similar classes of minerals.

## 1. Introduction

In nature, crystalline calcium carbonate (CaCO_3_) has three main polymorphic variants: calcite, aragonite, and vaterite. The stable field pressure–temperature phase diagram of CaCO_3_ shows that calcite is more stable under standard pressure and temperature conditions, while aragonite is more common at higher pressure and temperature values [1,2]. Vaterite, whose formation is controlled by dynamic factors, is a thermodynamically metastable phase that rapidly transforms into calcite or aragonite under certain conditions [3]. However, metastable vaterite can exist with a higher stability and resilience in the presence of organic molecules. For example, stable vaterite crystals have been found in green turtle eggshells [4], in coho salmon otoliths [5], and in the aberrant growth of mollusk shells repaired after an injury [6]. Han et al. [7] have found that thermoresponsive poly(N-isopropylacrylamide) brushes with a thickness of about 500 nm can act as effective polymer matrices for tuning the orientation and morphology of vaterite thin films along the *c* axis. Therefore, understanding the growth mechanism and pathway of vaterite in the presence of organic molecules is a key step in advancing and optimizing the manufacture of CaCO_3_ products for commercial applications.

In general, factors including solvent, temperature, stirring speed, pH of the medium, ionic concentration, and inorganic or organic additives could affect the morphology or size of vaterite particles in in vitro experiments. For instance, Yao et al. reported a series of highly monodispersed vaterite microspheres synthesis using water or dimethylformamide (DMF) as the solvent [8]. By mixing CaCl_2_ and Na_2_CO_3_ at pH 10, spheroidal vaterite grains with a diameter of 25–35 nm are obtained [9]. Andreassen and Hounslow added NaNO_3_ to separate solutions of Ca(NO_3_)_2_ and Na_2_CO_3_ and mixed the two solutions in equal volume to obtain vaterite pellets [10]. Flaten et al. [11] reported vaterite crystal growth in mixed ethylene glycol (EG) water solvent with different EG mass fraction at conditions of gas processing. The results showed that EG reduces the growth rate constant of vaterite when the reaction is second order. Spheroidal vaterite composed of 70 nm irregular nanoparticles have been synthesized via a fast microwave-assisted method in EG-water solvents, the ratio of EG to water, microwave power, reaction time, and ammonium ions are key parameters for spheroidal vaterite fabrication [12]. The influence of polyols (EG, glycerol, and erythritol) are studied with respect to their concentration, viscosity of water/polyol mixtures, and the number of hydroxyl groups per polyol molecule. Among them, EG and glycerol are found to promote the formation of vaterite enabling spherical framboid particles with the size from 2 μm to 350 nm [13]. Hollow vaterite microspheres with about 800 ± 100 nm diamete ion were successfully precipitated by adding calcium acetate and NaHCO_3_ to EG [14]. In these processes, the presence of EG changes both the solubility and the rate of crystal growth of vaterite particles. However, the preparation of a complex hierarchical structure, especially a monolayer and multilayer flower-like morphology of vaterite polycrystalline, which may be potentially applied as a smart container for various personal care or biomedical applications, has not previously been investigated.

In this work, the synthesis of metastable vaterite with a hierarchical structure was controlled by choosing suitable organic additives and employing a rapid microwave heating process. The morphology of vaterite could be regulated in a monolayered and multilayered flower-like scaffolding by changing the reaction conditions. The analysis of a vaterite microstructure shows that there are two different kinds of crystal cells in the structure of such vaterite flower dendrites. The experimental results provide an effective way to control the synthesis of calcium carbonate materials with hierarchical structures.

## 2. Experimental Section

CaCl_2_·2H_2_O, ethylene glycol (EG), and Na_2_CO_3_ analytical reagents were purchased from Sinopharm chemical reagent Shanghai Co. Ltd. (Shanghai, China), and 2-naphthoxyacetic acid (2-NAA, MW = 202.21, 98%) was purchased from ACROS Organics. Deionized water (18.25 MΩ·cm) was used in all experiments.

A 900 W Midea brand household microwave oven was used as the reactor after being equipped with a reflux device. The microwave was run in 22-s circulation mode for all experiments with open and stop times of 17 s and 5 s, respectively. The microwave power could be adjusted according to accommodate several power settings, including 270 W, 630 W, and 900 W. 

In a typical synthesis process, 0.202 g (0.001 mol) of 2-NAA and 1 mL of ethylene glycol (EG) were added to a 100-mL round-bottomed flask and ultrasonically treated to form a transparent solution. A 44 mL solution containing 0.294 g (0.002 mol) of CaCl_2_·2H_2_O was then added, followed by 1 min of ultrasonic treatment, rapid injection of a 5 mL solution containing 0.212 g (0.002 mol) of Na_2_CO_3_, and 10 s of ultrasonic treatment. The final concentrations of Ca^2+^, CO_3_^2^^−^, and 2-NAA were 40, 40, and 20 mM, respectively, the volume fraction of EG was 2% in aqueous solution. The flask was then placed in the microwave reactor and heated at a microwave power of 270 W, 630 W or 900 W. After heating, the flask was naturally cooled to room temperature, the supernatant was discarded, and the white precipitate was washed three times each with distilled water and ethanol and then vacuum dried. All experiments were carried out following this protocol.

The mineral phases and structures were detected using a Rigaku X-ray diffractometer (XRD, Cu, Kα, λ = 0.154056 nm) and a Nicolet Instrument MAGNA-IR 750 Fourier-transform infrared spectrometer (FT-IR). An FEI Sirion-200 field emission scanning electron microscope (FESEM) was used to observe the mineral morphologies. Transmission electron microscopy (TEM) images, selection area electron diffraction (SAED) patterns, and high-resolution electron microscopy (HRTEM) images were obtained under a JSM JEOL-2010 microscope with an acceleration voltage of 200 kV.

## 3. Results

**Figure 1:**
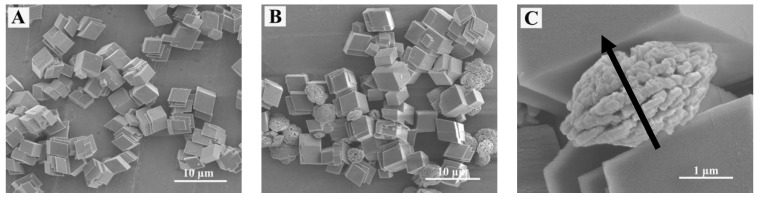
The FESEM images obtained after 10 min from an aqueous solution (**A**) and an queous solution containing EG (**B**,**C**).

**Figure 2:**
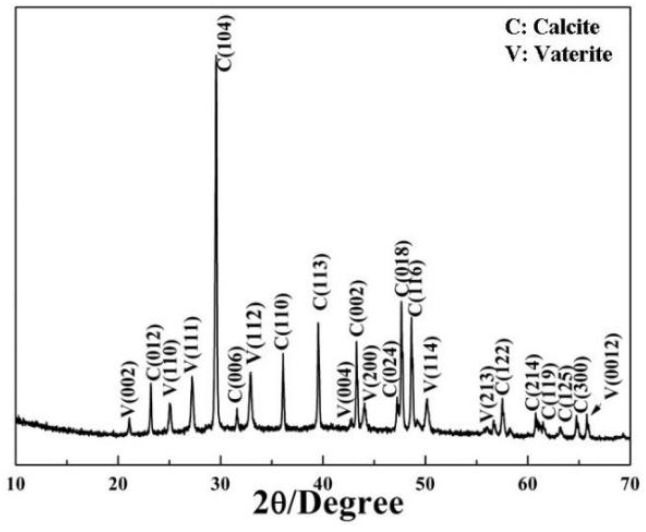
The XRD pattern of the product obtained from the reaction system containing 1 mL of EG.

Figure 1A shows the FESEM image of the sample obtained without adding EG or 2-NAA. It can be clearly seen that all particles only show the typical rhombohedral morphologies of calcite [15]. However, a small amount of flat disc-like particles appeared when 1 mL of EG was added to the reaction pot (Figure 1B). The average diameter and thickness of the disc-like particles were 3 and 1.5 μm, respectively. The enlarged image showed that these disc particles were orderly constructed from smaller particles with an average diameter of 200 nm when measured in one orientation as indicated by the black arrow in Figure 1C). The XRD pattern in Figure 2 shows that the major product was calcite (JCPDS No. 72-1214), with a small amount of vaterite (JCPDS No. 72-0506). As calcite crystals usually exhibit a rhombohedral morphology, it was reasonable to assume that the disc-like particles were vaterite, indicating that the addition of EG was conducive to a metastable vaterite formation.

**Figure 3:**
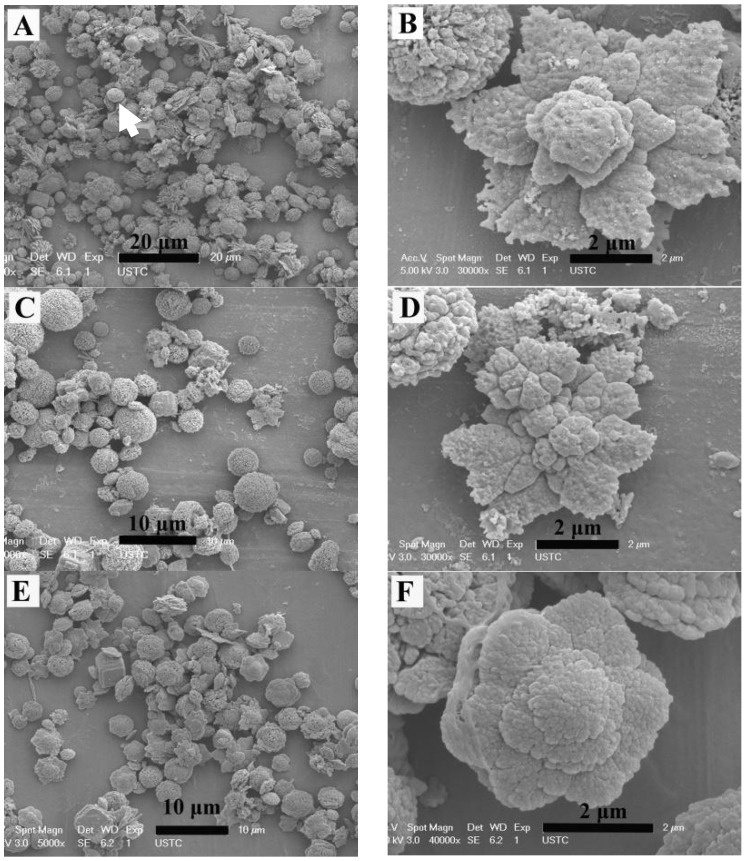
The FESEM images of calcium carbonate obtained at different microwave powers. Microwave power: (**A**,**B**) 270 W, (**C**,**D**) 630 W, (**E**,**F**) 900 W.

**Figure 4:**
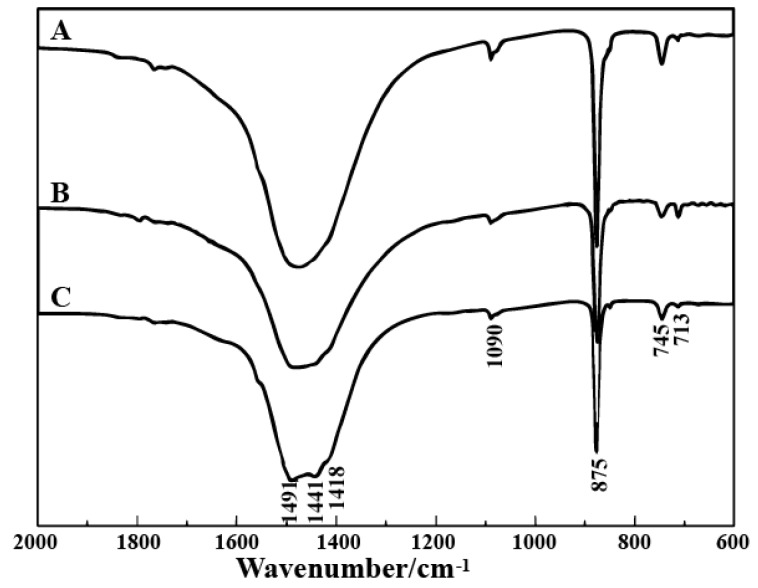
The FT-IR spectra of CaCO_3_ obtained at different microwave powers. (**A**) 270 W, (**B**) 630 W, and (**C**) 900 W.

When 0.202 g 2-NAA was added to the pot and the microwave power was set at 270 W for 10 min, the products exhibited rhombohedral, spherical, and flower-like morphologies (Figure 3A). The spherical particles were composed of nano-sized spherulites and their average diameter was 3 μm (indicated by the white arrow in Figure 3A). A typical flower-like crystal is shown in Figure 3B, which clearly highlights the six petals growing radially from the center in the same plane, with smaller petals staggered on the lower counterparts, rather than superimposing in the same direction in a vertical position. As the microwave power increased to 630 or 900 W, the obtained particles also showed rhombohedral, globular, and flower-like morphologies (Figure 3C,E). The typical flower-like particles are presented in Figure 3D,F, which were similar to the ones obtained at 270 W. Figure 4 shows the FT-IR spectra of the samples, and the peaks at 713 cm^−1^ (ν_4_), 875 cm^−1^ (ν_2_), and 1418 cm^−1^ (ν_3_) correspond to the in-plane bending vibration, the out-plane bending vibration, and the antisymmetric stretching vibration of calcite, respectively. The 745 cm^−1^ (ν_4_), 1080 cm^−1^ (ν_1_), and 1490 cm^−1^ (ν_2_) and 1440 cm^−1^ (ν_3_) splitting peaks correspond to the in-plane bending vibration, symmetric stretching vibration, and anti-symmetric stretching vibration absorption peak of vaterite [16]. The rhombohedral crystals could be identified as calcite [15]. The most common morphology of vaterite is spherical-like, composed of small particles [17,18], so we assumed that the spherical particles were vaterite. In the following discussion, the flower-like crystallites are also confirmed to be vaterite. The above results indicate that adding 2-NAA encouraged the formation of metastable vaterite.

**Figure 5:**
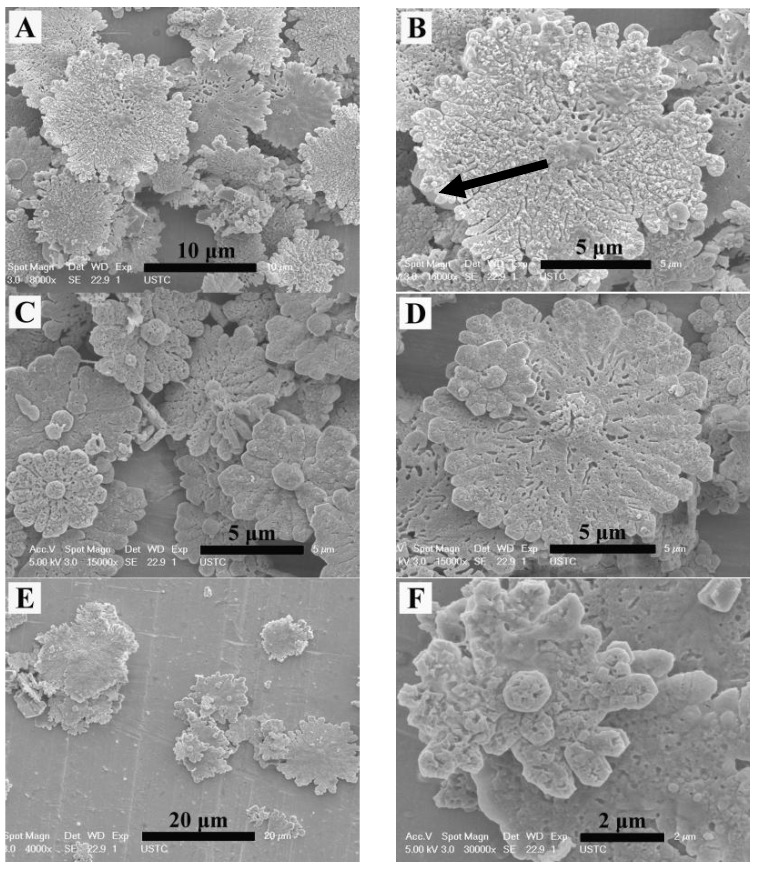
The FESEM images of vaterite obtained by adding EG and 2-NAA to the reaction system. Reaction time: (**A**,**B**) 3 min, (**C**,**D**) 5 min, and (**E**,**F**) 10 min.

**Figure 6:**
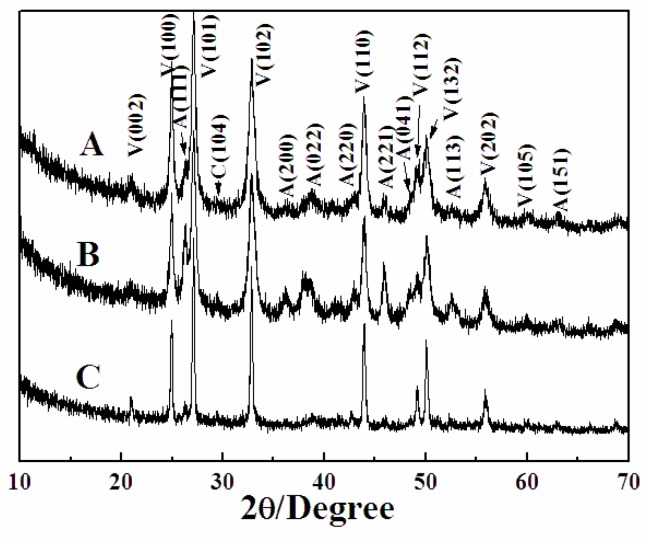
The XRD patterns of CaCO_3_ obtained after adding EG and 2-NAA to the reaction pot. Reaction time: (**A**) 3 min, (**B**) 5 min, and (**C**) 10 min. V, A, and C represent vaterite, aragonite, and calcite, respectively.

When 1 mL of EG was added to dissolve 2-NAA and the microwave exposure was set to 3 min, the obtained particles exhibited a mostly flower-like morphology, with a small amount of rod-like and rhombic morphology (Figure 5A). The surface of the flower-like crystal was rough and uneven with irregular edges. An amplified image shows that the growth radiates from the center, as indicated by the black arrow in Figure 5B. In order to explore the evolution of crystal morphology with time, the experiment was prolonged to 5 min. FESEM images (Figure 5C,D) showed that a spherical bud emerged from the center of the flower-like crystal, indicating that growth started from the center of the flower-like crystal. When the experimental time was further prolonged to 10 min, FESEM images (Figure 5E,F) showed that the flower-like morphology was preserved. XRD patterns shown in Figure 6 revealed that the majority (93%) of the samples were vaterite (JCPDS No. 72-0506) when EG and 2-NAA were added even at differing reaction times, suggesting that the flower-like crystals in Figure 5 were vaterite, despite its distinction from the usual spherical morphology of vaterite. Furthermore, a small amount of aragonite (JCPDS No. 05-0453) and calcite (JCPDS No. 72-1214) were also observed to coexist. 

**Figure 7:**
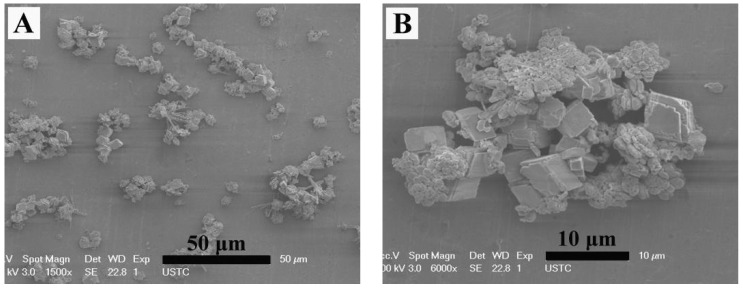
(**A**) The panoramic and (**B**) amplified FESEM images of CaCO_3_ crystals obtained after adding EG and 0.101 g 2-NAA to the reaction system.

**Figure 8:**
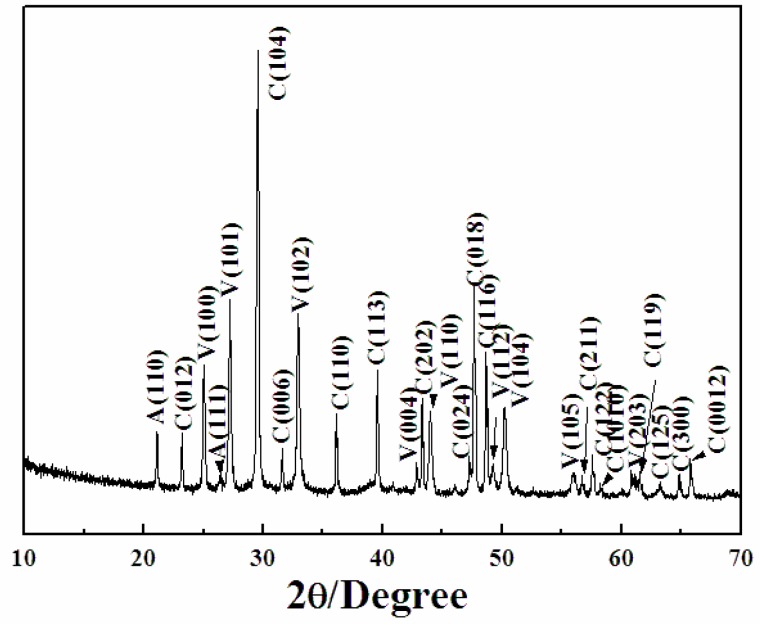
The XRD pattern of CaCO_3_ obtained after adding 0.101 g 2-NAA to the reaction pot. V, A, and C represent vaterite, aragonite, and calcite, respectively.

To investigate the influence of 2-NAA on the amount on the phase and morphology of CaCO_3_, only 0.101 g of 2-NAA was added to the reaction pot. Figure 7 shows that in addition to a large number of rhombohedral particles, a small number of particles without a uniform morphology and size emerged, and no flower-shaped crystals appeared in this product. The XRD pattern in Figure 8 showed that the main phase of the product was calcite (JCPDS No. 72-1214), with small amounts of vaterite (JCPDS No. 72-0506) and aragonite (JCPDS No. 05-0453).

**Figure 9:**
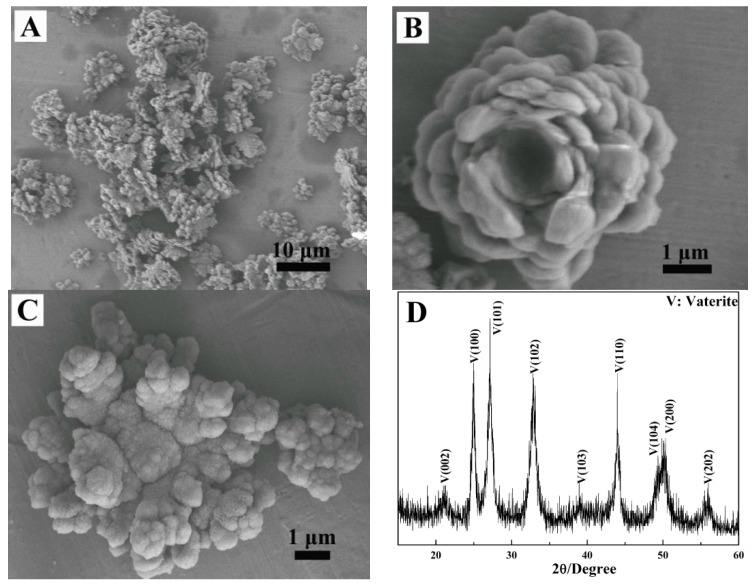
The FESEM images (**A**–**C**) and XRD (**D**) pattern of CaCO_3_ obtained after adding 0.404 g of 2-NAA. Reaction time: 5 min.

**Figure 10:**
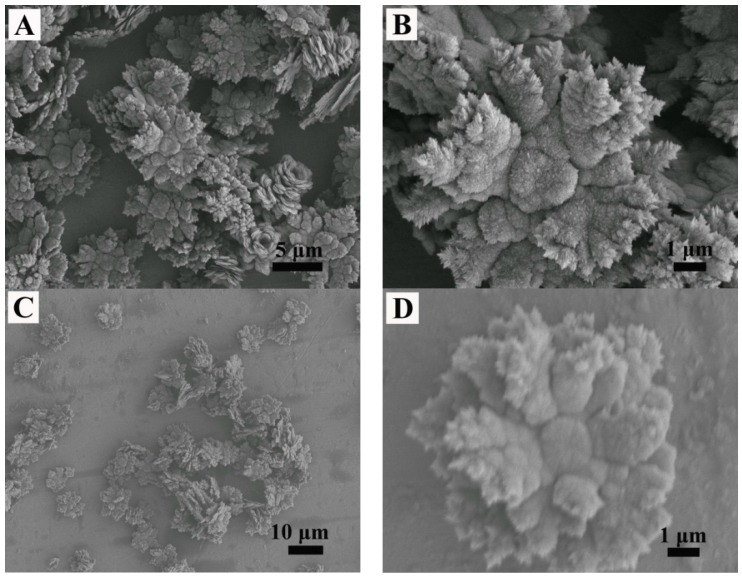
The FESEM images of CaCO_3_ crystals obtained after adding 0.404 g of 2-NAA to the reaction pot. Reaction time: (**A**,**B**) 10 min, (**C**,**D**) 30 min.

When 0.404 g of 2-NAA was added to the reaction pot, a rosette-like morphology of CaCO_3_ appeared (Figure 9A), and Figure 9B,C present two typical rosette-like morphologies. The XRD pattern (Figure 9D) revealed that the obtained sample was in a pure vaterite phase (JCPDS No. 72-0506). The half-high width of the diffraction peak (102) was measured to be 0.0119 *Rad* and the average grain size of the flower-like vaterite calculated by the Scherrer formula was approximately 12 nm, which suggests that the flower-like vaterite crystals were composed of tiny nano-sized grains. If the reaction time was further prolonged to 10 min (Figure 10A), the obtained particles still presented a flower-like morphology. Compared to the particles obtained after 5 min (Figure 9A), the petals grown around the center and stacked layer upon layer presented a perfect flower-like shape (Figure 10B). With the reaction time being prolonged to 30 min, the crystal morphologies were almost the same as those obtained after 10 min (Figure 10C,D). Compared to the samples obtained when adding 0.101 g or 0.202 g of 2-NAA to the reaction pot, the crystal phases and morphologies are obviously different, which indicated that the addition of 2-NAA significantly affected the phase and morphology of the product.

## 4. Microstructure Analysis of Flower-Like Vaterite Crystals

**Figure 11:**
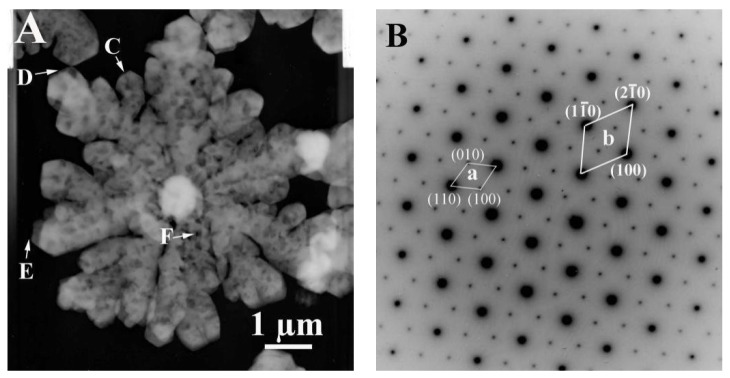

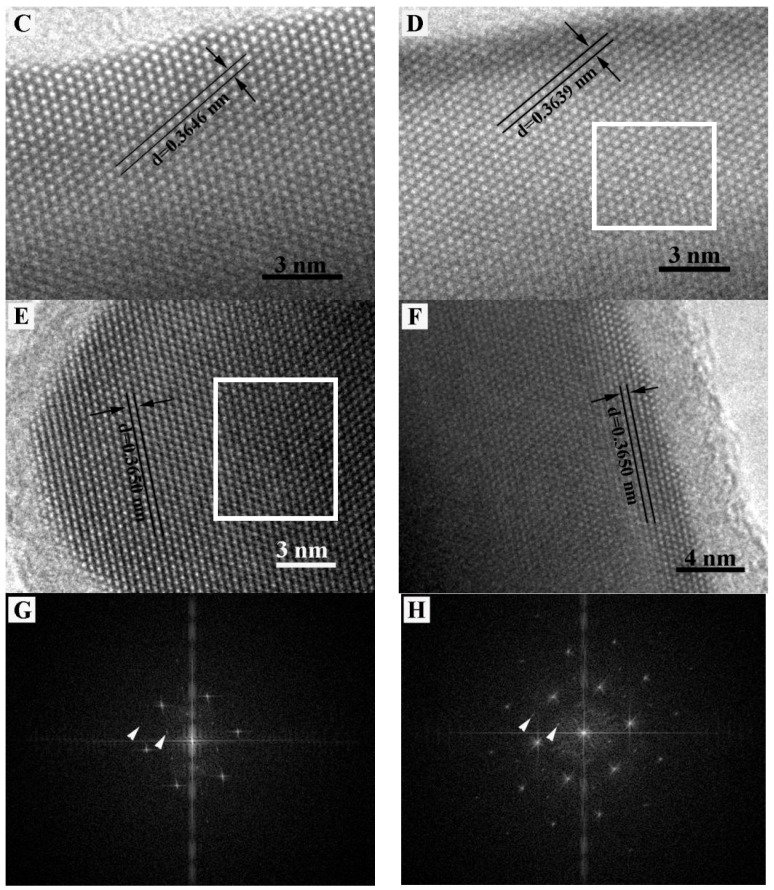
The microstructure analysis of a typical flower-like vaterite crystal. (**A**) TEM image, (**B**) SAED pattern, (**C**–**F**) HRTEM images of **C**–**F** labeled points in Figure (**A**), (**G**,**H**) FFT images of the box areas of (**D**,**E**).

**Figure 12:**
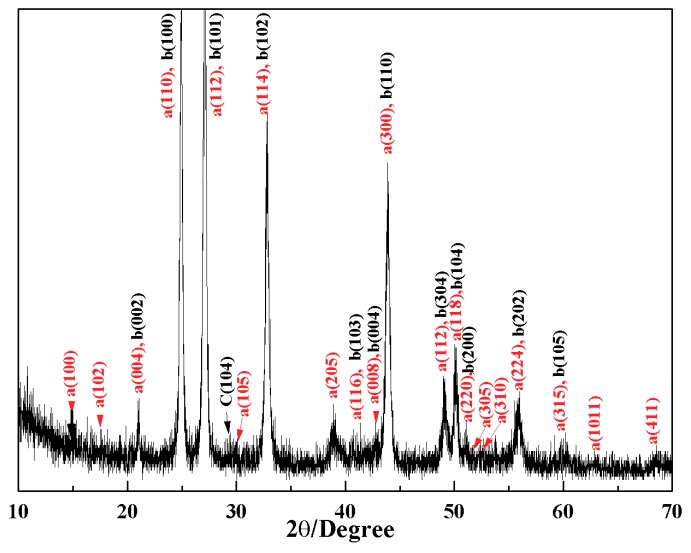
The XRD pattern of the sample obtained after 10 min.

**Figure 13:**
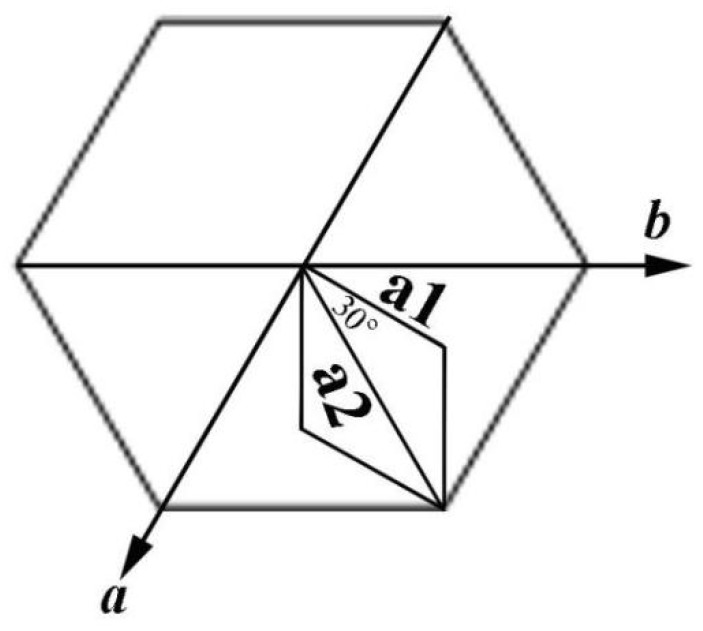
The relationship between two sets of crystal cell parameters by a_2_ = 2a × cos30°, with the *c* axis direction being perpendicular to the principal plane.

The crystal microstructure shown in Figure 5D that showed a typical flower-like crystal adorned with a globular pistil was further analyzed using TEM, HRTEM, and SAED. Figure 11A shows the TEM image of a complete crystal. It can be seen that the crystal extends radially outward from the central position like petals and has six symmetrical structures. The SAED pattern in Figure 11B of the entire flower-like crystal shows a perfect single crystal structure. These spots could be characterized as (100), (010), and (110) diffraction of vaterite (JCPDS no.72-1212), and the crystallographic zone axis analyzed by SAED was [001]. The corresponding crystal cell parameters were a_1_ = b_1_ = 7.147 Å, and c_1_ = 16.989 Å, and the space group was *P63/mmc*, marked “a” in Figure 11B. These results were inconsistent with the XRD pattern in Figure 12. In the XRD pattern, all the peaks could be denoted as a larger set of cell parameters (a_1_ = b_1_ = 7.147 Å, and c_1_=16.989 Å, JCPDS No. 72-1212) of vaterite (marked “a”). A subset of the diffraction peaks could also be denoted as a set of small crystal cell parameters (a_2_ = b_2_ = 4.130 Å and c_2_ = 8.490 Å, JCPDS No. 72-0506) of vaterite (marked “b”). Some diffraction peaks corresponding to these two crystal cell parameters may appear at the same peak position. These results indicate that two types of vaterite with different crystal cell parameters coexisted in the same crystal. This was also confirmed by SAED patterns. In the SAED pattern shown in Figure 11B, the stronger diffraction spot can also be marked as (100), (1–10), and (2–10) crystallographic planes of vaterite (JCPDS No. 72-0506), with corresponding crystal cell parameters of a_2_ = b_2_ = 4.13 Å and c_2_ = 8.490 Å, and a space group of *P63/mmc*, marked “b” in Figure 11B. This also indicated that two types of cells (a and b) grew coherently within the same flower-like crystal. The quantitative relationship between the two sets of crystal cell parameters was determined by a_1_ = 2a_2_ × cos30° and c_1_ = 2c_2_, as shown in Figure 13. HRTEM observation at different positions (marked as C, D, E, and F) on the flower-like crystal in Figure 11A are shown in Figure 11C–F. The uniform lattice fringes indicate that the crystal has a single crystal structure. At four different positions, the lattice spacing between adjacent crystal faces was 0.3646 nm, 0.3639 nm, 0.3650 nm, and 0.3650 nm, which was consistent with the set of smaller cell b (a1 = b1 = 4.130 Å and c1 = 8.490 Å, JCPDS No. 72-0506). It is also consistent, however, with the (100) lattice plane (d = 0.3577 nm) of vaterite with the larger crystal cell a (a_2_ = b_2_ = 7.147 Å and c_2_ = 8.490 Å, JCPDS No. 72-1616). The fast Fourier-transform (FFT) images of the square region in Figure 11C,D are shown in Figure 11G,H. It can be seen that each of the FFT images has two sets of diffraction spots with different diffraction intensity, and the weaker spots are shown in Figure 11G,H with white arrows. This also illustrates that a larger set of crystal cell a (JCPDS No. 72-1212) and a smaller set of cell b (JCPDS No. 72-0506) coexist in the same vaterite crystal. For the nature of vaterite structure, Demichelis et al. [19,20] have reported at least three effectively isoenergetic models on the basis of ab initio calculations. Their results demonstrate that vaterite is not a single “disordered” structure but should instead be considered as a combination of different forms, each of which can exhibit rapid interchange between multiple structures that exhibit similar average properties. Interestingly, Kabalah-Amitai et al. [21] findings from the body and tunic spicules of the solitary stolidobranch ascidian *Herdmania momus* also indicate that the vaterite crystal structure is not a single entity, rather, it is actually composed of at least two different crystallographic structures that coexist within a pseudo–single crystal. 

## 5. Discussion

Li et al. [22] reported that spherical, plate, sheet, and almond-shaped vaterite could be synthesized with a solvent-thermal method using EG, 1,2-propylene glycol, or glycerin as the solvents but not in an aqueous solution. Therefore, it seems likely that polyalcohols, such as EG, 1,2-propylene glycol, and glycerin, stabilize vaterite crystal nuclei via the electronegative hydroxyl groups. This adsorption changes in the surface energy of vaterite stabilize the vaterite crystal nuclei and allow the formation of metastable vaterite crystals. The {001} plane of vaterite is composed of calcium ions or carbonate ions and alternates so that it is either positively or negatively charged [23]. With rapid microwave heating, EG molecules were adsorbed onto the vaterite crystal surface via the electronegative hydroxyl group, reduce the surface energy of the crystal, and then stabilize the disc-like vaterite (Figure 1B,C). However, due to the small amount of EG (V_EG_:V_H2O_ = 1:49), EG could not stabilize all the vaterite crystals and a large number of calcite phase crystals coexisted (Figure 1 and Figure 2). When the EG amount was increased, for example to V_EG_:V_H2O_ = 49:1, Figure 14A shows that almost all vaterite crystals possessed flower-like morphologies, with a very small presence of other phases such as calcite and aragonite.

In general, metastable vaterite structures with various morphologies are composed of nanometer-sized grains that accumulate in different patterns [13,24,25,26]. When 2-NAA was added to the reaction pot as a mineral regulator, it stabilized the vaterite {001} face with its electronegative carboxyl, resulting in a flower-like vaterite, an effect similar to the polyhydroxyl group of EG. However, because of the poor solubility of 2-NAA in aqueous solution, it may not be sufficient to stabilize all vaterite crystals, a spherical and flower-like vaterite mixed with rhombohedral calcite was obtained. When EG was added, 2-NAA dissolved and the effect of EG carboxyl and 2-NAA carbonate stabilized the high-energy {001} face of the vaterite crystal, resulting in the formation of flower-like vaterite. Increasing the reaction time encouraged the further nucleation of vaterite on the center of the generated flower-like crystals, causing extension in three dimensions and forming a blossom on the flower-like crystals (Figure 5).

This microstructure analysis of the flower-like vaterite crystals shows that they have dendritic structures. A variety of different materials with dendritic structure have been reported in the literature, such as nano-structured PbS [27], the pine-tree branches of a-Fe_2_O_3_ [28], platinum nanoparticles [29], and palladium and silver [30], However, metastable vaterite with a flowering dendritic structure had not previously been described in the literature. The formation process of vaterite flower-like dendritic structure in this experiment could be explained as follows. Vaterite crystals with hexagonal structure have [100] faces in six equivalent directions. During crystal formation, if one of the directions, for example, the [100] direction nucleates first and has a faster growth rate, an acyclic crystal will form at the beginning. Then, this acyclic crystal will grow along the two isovalent crystallographic directions, namely [1–10] and [0–10], to form symmetrical bifurcation. All the bifurcations will then thicken and eventually connect to each other as they continue to grow. The growth rate of vaterite crystals along six symmetry directions (±[100], ±[1–10], and ±[0–10]) is, in fact, equivalent; therefore, the formation of a flower-like morphology with six-fold symmetry is the inevitable result [28]. It is important to note that because the {001} face of vaterite is composed of alternating calcium and carbonate ions, it will be positively or negatively charged. In this experiment, the electronegativity of the carboxyl groups of 2-NAA neutralized the ionic charge of the polar surface, therefore inhibiting growth along the [001] direction. Thus, the formation of flower-like vaterite parallel to (001) was the most probable option.

## 6. Conclusions

In this work, dendritic vaterite crystals with a flower-like morphology were synthesized using a simple microwave heating method. The results indicated that a small amount of EG and 2-NAA could effectively regulate the formation of dendritic flower-like vaterite. During the formation process of this flower-like vaterite, the electronegativity of 2-NAA significantly neutralized the charge of the vaterite (001) plane, reducing the polar face energy and resulting in crystal growth along the direction parallel to the (001) plane. The microstructure analysis of this flower-like vaterite shows that its crystals were composed of two types of cells of different sizes. This new understanding on the influence of carbonate provides insights into the complex and unsuspected effect of organic molecules on CaCO_3_ crystallization and the mechanisms used by organisms to design fit-for-purpose biominerals and may provide guidance for shape- and size-controlled fabrication of CaCO_3_ matrices with complex mineral structures.

## Figures and Tables

**Figure 14:**
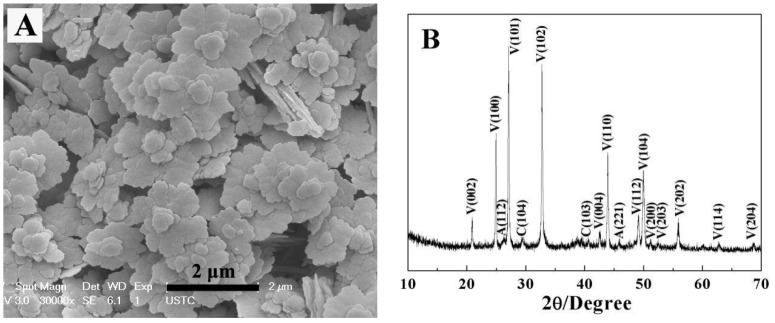
The FESEM image (**A**) and XRD pattern (**B**) of CaCO_3_ obtained with V_EG_:V_H2O_ = 49:1.
